# Determinants of the Empiric Use of Antibiotics by General Practitioners in South Africa: Observational, Analytic, Cross-Sectional Study

**DOI:** 10.3390/antibiotics11101423

**Published:** 2022-10-17

**Authors:** Sinenhlanhla Pearl Guma, Brian Godman, Stephen M. Campbell, Ozayr Mahomed

**Affiliations:** 1 Discipline of Public Health Medicine, 227 George Campbell Building, Howard College Campus, University of KwaZulu-Natal, Durban 4001, South Africa; 2 Department of Public Health Pharmacy and Management, School of Pharmacy, Sefako Makgatho Health Sciences University, Pretoria 0204, South Africa; 3 Centre of Medical and Bio-allied Health Sciences Research, Ajman University, Ajman P.O. Box 346, United Arab Emirates; 4 Department of Pharmacoepidemiology, Strathclyde Institute of Pharmacy and Biomedical Science (SIPBS), University of Strathclyde, Glasgow G4 0RE, UK; 5 Centre for Epidemiology and Public Health, School of Health Sciences, University of Manchester, Manchester M13 9PL, UK; 6 NIHR Greater Manchester Patient Safety Translational Research Centre, School of Health Sciences, University of Manchester, Manchester M13 9PL, UK

**Keywords:** empiric antibiotic prescribing, general practitioners, antimicrobial resistance, South Africa, diagnostic uncertainty, guidelines

## Abstract

The overuse of antibiotics is the main driver of antimicrobial resistance (AMR). However, there has been limited surveillance data on AMR and antibiotic prescribing at a primary healthcare level in South Africa. An observational, analytic, cross-sectional study was undertaken to assess key factors associated with empiric antibiotic prescribing among private sector general practitioners (GPs) in the eThekwini district in South Africa, particularly for patients with acute respiratory infections (ARIs). A semi-structured web-based questionnaire was used between November 2020–March 2021. One hundred and sixteen (55.5%) responding GPs prescribed antibiotics empirically for patients with ARIs more than 70% of the time, primarily for symptom relief and the prevention of complications. GPs between the ages of 35–44 years (OR: 3.38; 95%CI: 1.15–9.88), >55 years (OR: 4.75; 95% CI 1.08–21) and in practice < 15 years (OR: 2.20; 95%CI: 1.08–4.51) were significantly more likely to prescribe antibiotics empirically. Three factors—workload/time pressures; diagnostic uncertainty, and the use of a formulary, were significantly associated with empiric prescribing. GPs with more experience and working alone were slightly less likely to prescribe antibiotics empirically. These findings indicate that a combination of environmental factors are important underlying contributors to the development of AMR. As a result, guide appropriate interventions using a health system approach, which includes pertinent prescribing indicators and targets.

## 1. Introduction

Antimicrobial resistance (AMR) is a major public health challenge [1,2,3,4,5], with an estimated 1.27 million deaths globally in 2019 due to bacterial AMR, with up to 4.95 million deaths associated with bacterial AMR in 2019 [3]. The greatest burden of AMR is currently in sub-Saharan Africa [3]. The costs associated with AMR can be considerable, with the World Bank estimating that the loss of worldwide productivity arising from AMR after 2030 could exceed USD 1 trillion annually or greater, which is equivalent to 3.8% of annual Gross Domestic Product or more [6].

The overuse and misuse of antibiotics are the principal drivers of AMR [7,8,9,10], exacerbated by increasing inappropriate utilization of antibiotics among low- and middle-income countries (LMICs) [11]. Overall, in LMICs, up to 80% of antibiotic consumption among patients is at the community level, of which 20–50% or more is seen as inappropriate [12,13,14]. Some studies have suggested that for certain conditions in LMICs, inappropriate prescribing can reach 100% [15]. A considerable proportion of antibiotics are prescribed inappropriately for acute respiratory tract infections (RTIs), which are essentially viral in origin requiring no antibiotic to aid symptom relief [7,16,17,18,19]. There are a number of factors that influence prescribing behavior. These include social-cultural and socio-economic factors, diagnostic uncertainty, clinical competency, cultural beliefs of the patient, patient demand, time pressures on physicians and patients as well as clinical autonomy [16,20,21,22,23,24].

Key issues and concerns regarding the rising rates of AMR globally, and the subsequent consequences, have resulted in the World Health Organisation urging countries, including African countries, to develop their national action plans (NAPs) to tackle AMR [25,26,27]. South Africa is no exception [28], building on the South African Antimicrobial Resistance National Strategy Frameworks as well as ongoing activities to encourage antimicrobial stewardship programmes (ASPs) starting in hospitals [28,29,30]. The South African Antimicrobial Resistance National Strategy Framework 2014–2024 consisted of three main pillars. These were firstly surveillance of antimicrobial resistance data and antibiotic use; secondly infection prevention control and lastly antimicrobial stewardship [30]. However, there is currently limited data nationally regarding antimicrobial use in ambulatory care among LMICs, which is exacerbated by the considerable purchasing of antibiotics without a prescription that still takes place in a number of these countries [10,31,32,33].

A key element of South African and other NAPs is the documentation of current antimicrobial usage and resistance patterns across all sectors of care [27,28,34]. We are aware that there have been a number of studies undertaken in South Africa to monitor prescribing and resistance patterns in ambulatory care; however, these have shown variable findings alongside requests for additional data to guide future strategies [14,24,35,36,37]. In addition, there have been concerns among physicians in South Africa regarding their knowledge of antibiotics and AMR as well as confidence with prescribing antibiotics [38]. Between 2003 and 2005, two pilot projects undertaken in KwaZulu-Natal (KZN) Province and Brits (North West Province) assessed antibiotic use and resistance. These pilot projects showed high inappropriate use of antibiotics in KZN and North West Province; however, they did not comment on the reasons or factors that might be associated with the prescribing patterns seen [39]. Farley et al. (2018) found that whilst 95.8% of primary care physicians in the private sector believed that AMR was a significant problem in South Africa, two thirds of those surveyed felt pressure from patients to prescribe an antibiotic when visited [24]. Alongside this, Gasson et al. (2018) found poor adherence to national antibiotic prescribing guidelines amongst primary care physicians in the public sector in Cape Town [14], with Matsitse et al. (2017) finding similar concerns among healthcare professionals (HCPs) treating patients with sexually transmitted diseases in the public sector in South Africa [40]. Ncube et al. (2017) also demonstrated high rates of antibiotic prescribing among physicians treating private patients with acute bronchitis [41], with Manderson (2020), Mathibe et al. (2020) and others documenting similar concerns among physicians treating both private and public patients with acute respiratory infections [42,43,44]. However, Skosana et al. (2022) in their study showed high adherence rates to South African guidelines (93.4%) when HCPs prescribed antibiotics for patients attending community health centres, with ear, nose and throat infections the most common presentation [37]. 

We wanted to build on these findings by ascertaining key healthcare provider and environmental factors currently influencing empiric antibiotic prescribing among GPs in South Africa starting in the private sector. In this case, empiric prescribing of antibiotics is based on the clinical presentation, past experiences and workplace issues, i.e., typically based on experience and a ‘best guess’ for the intention of treating potential infectious diseases. However, often in the absence of clear evidence or with diagnostic uncertainty and without knowledge of the cause [45]. The findings from our study can be used to develop appropriate strategies, including agreed prescribing targets, to improve future antibiotic prescribing in ambulatory care in South Africa and wider. We believe this is important given increasing global concerns regarding AMR especially across Africa [3,10,27]. Table 1 documents key prescribing indicators, including quality indicators, used across countries to improve antibiotic prescribing in ambulatory care building on recent reviews [46]. However, it is important that there are systems in place to monitor performance especially if agreed indicators include a confirmed diagnosis. For instance in Scotland, the healthcare system currently only collects data on medicines dispensed in ambulatory care and not the indication; this has to be inferred from hospital data aided by patients having the same healthcare number unless other data sets are available [47,48,49]. This compares with Botswana and Sweden where diagnostic data is available in ambulatory care in their healthcare systems to audit the prescribing performance of GPs [50,51,52].

These suggested developments will build on the current national action plan to reduce AMR in South Africa, including suggested strategies to reduce high rates of AMR [27,28,74]. This is imperative at this time given growing concerns across countries with high rates of inappropriate use of antibiotics in ambulatory care generally as well as for patients with COVID-19 despite limited evidence of bacterial or fungal co-infections [75,76,77,78]. Consequently, the objectives of this study were to identify the prevalence of, and contributory factors, behind current empiric prescribing of antibiotics by GPs in South Africa starting with private GPs. This is because we are aware there can be different prescribing practices between physicians working in the private versus public sectors, with often greater pressure and expectations from patients or their parents for physicians in the private sector to prescribe antibiotics [16,22,79]. 

## 2. Materials and Methods

### 2.1. Study Design and Population

The study design was an observational, analytic, cross-sectional design performed with data collected from primary care physicians from November 2020 to March 2021. Overall, there were 1160 GPs in the private sector in this district. The inclusion and exclusion criteria are documented in Table 2.

We chose to concentrate on the private sector for this study as we were aware that previous studies in South Africa had shown concerns with the prescribing habits of GPs in the private sector [41,42,43,44]. As mentioned, there have been concerns in other LMICs regarding the extent of inappropriate prescribing of antibiotics among physicians working in the private versus public sectors [22,79,80], and the private sector accounts for a significant minority of patients in South Africa [81]. However, there have been ongoing activities by the Government in South Africa and other key stakeholder groups in South Africa in recent years to reduce inappropriate use of antibiotics across sectors [27]. This is reflected by appreciably greater adherence to guidelines among public facilities in ambulatory care compared with the previous situation seen among both private and public ambulatory care facilities [14,37,40,41,42].

### 2.2. Study Variables and Sample Size

The dependent variable was the empiric prescribing of antibiotics. The independent variables included all socio-demographic independent variables, i.e., age, gender, type of practice, number of years in private practice, dispensing, number of consultations per day, estimated number of antibiotics prescribed in the last week, attended antibiotic training courses, attended relevant international conferences, lack of antibiotic prescribing guidelines, presence of comorbidities, patient expectation/requests, and concerns with AMR. Adherence to robust guidelines is increasingly seen as a marker of the quality of prescribing across sectors among countries including African countries [10,14,46,82,83,84,85,86,87]. 

The sample size was calculated using Yamene’s formula: n = N/1 + N (e)^2^ using a significance level of 0.05 and confidence level of 95%, where n is a sample size, N is the population size (the universe), e is a sampling error (usually 0.10, 0.05 and 0.1 acceptable error) [88]. The sample size, calculated using StatCalc sample size and a power calculator from the total number of GPs in the eThekwini District, i.e., 1160, yielded 252 for 95% CI, 5% acceptable margin of error. 366 GPs were subsequently invited.

### 2.3. Questionnaire Design and Analysis

A semi-structured web-based questionnaire with closed-ended questions was developed based on a literature review of published studies with similar objectives combined with the knowledge and experience of the co-authors [24,42,89]. The main categories of the questions from the published international studies were used to develop context specific questions, although the questions from previous studies were not used verbatim but adapted for the local situation in South Africa. We have successfully used this approach before when developing qualitative questionnaires for specific situations [16,27,90,91,92,93,94]. 

To enhance the robustness of the questionnaire used in the principal study, a pilot study was undertaken involving 30 GPs. These GPs were subsequently excluded from the principal study. The questionnaire was further refined for clarity of language but not content before implementation of the principal study. As a result, adding validity to the findings and their implications. 

The final questionnaire was subsequently distributed through the medical professional associations. To increase the response rates, GPs were reminded telephonically and asked to participate in the survey. 

The final questionnaire addressed six broad areas. These were the socio-demographics of participants; possession of guidelines; attitudes/perception; prescribing conditions (type of disease) and choices on antibiotics; as well as factors influencing GPs’ antibiotic prescribing and knowledge. 

The completed questionnaires were subsequently checked for completeness and were coded with a unique numerical identifier to remain anonymous. The replies were subsequently captured onto a password protected electronic database—QuestionPro database, with the raw data cleaned and coded before data analysis. The raw data and log files were stored separately and backed up using an external hard drive. After a data storage period of 5 years, the electronic data will be securely destroyed. 

Data analysis was conducted using Stata Version 13.0 (Stat Corp.2013.Stata Statistical Software: Release 13, College Station, TX: StataCorp LP). The socio-demographic variables were summarized using appropriate summary measures for categorical and numerical data and displayed in a table. Socio-demographic profile, factors influencing antibiotics prescribing and knowledge outcomes were summarized using frequency distributions. Ten variables were used to assess GPs’ knowledge. Key environmental and other factors included the rationale for prescribing antibiotics, workload/time pressures, presence of co-morbidities, and guideline availability. A score of one was assigned for a correct answer and zero for incorrect answer. The total scores were subsequently summed for each participating GP. The median score was calculated from the total score across all participating GPs. A score of ≥8 was categorised as good knowledge. 

Bivariate analysis was initially conducted to identify potentially significant associations. Odds ratios were subsequently calculated, and the results were assessed for significance at *p*-values < 0.05. Multi-regression analyses were used to identify socio-demographic variables and prescribers/patient/environmental factors that could predict empiric prescribing of antibiotics. All variables were included in the multivariate and regression analyses. 

## 3. Results

Three hundred and sixty-six GPs were invited to participate, with 243 agreeing to participate. 209 GPs were finally included in the study as 34 were excluded since their questionnaires were incomplete. This yielded a 57% response rate. 

### 3.1. The Extent of Empiric Prescribing

**Figure 1:**
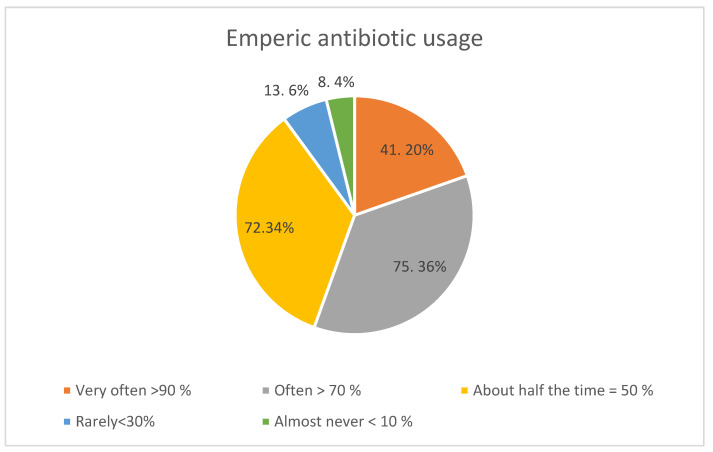
Proportion of respondents prescribing antibiotics empirically.

One hundred and sixteen (55.5%) of the GPs who completed the questionnaire indicated that they prescribed antibiotics empirically more than 70% of the time for presenting conditions, with a minority prescribing antibiotics empirically rarely or almost never (Figure 1). 

**Figure 2:**
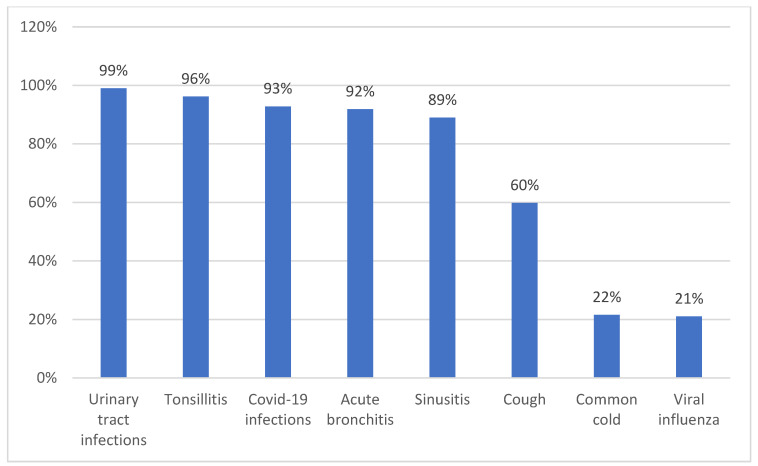
Proportion of respondents prescribing antibiotics empirically for common conditions for symptom relief and prevention of complications.

Regarding the potential quality of prescribing, participating GPs typically prescribed antibiotics for symptom relief and the prevention of complications for patients where urinary tract infections (UTIs) (99% of occasions) as well as acute sinusitis (89% of occasion) where antibiotics may well work. In addition, for essentially viral infections including tonsillitis (96%), COVID-19 infections (93%), and acute bronchitis (92%) as well as for coughs (60%) and colds where they are likely to have no effect on symptom relief (Figure 2).

### 3.2. Prescribing Patterns

Broad spectrum penicillins, e.g., amoxicillin, were the most commonly prescribed antibiotic against all conditions ranging from 12% of participating GPs for UTIs to 82% of physicians for tonsillitis, which is a clear misuse of antibiotics. Fluroquinolones, e.g., ciprofloxacin, were the most commonly prescribed antibiotics (81%) amongst participating GPs for patients diagnosed by the GPs as having UTIs, with very limited or no prescribing for respiratory infections including tonsilitis, influenza, common colds and COVID-19. Carbapenems were mostly prescribed for sinusitis (10% of participating GPs) and acute bronchitis. Combinations of penicillins were principally prescribed for patients with COVID-19 (42%).

### 3.3. Demographic Factors Associated with Empiric Antibiotic Prescribing

The majority of participating GPs who completed the questionnaire (170, 81.3%) were under 55 years of age, with males (58.4%) being the most predominant gender. 64.1% of GPs (134) were in solo practices with the majority (60.8%;127) being in practice for less than 15 years. 74.2% (155) of participating GPs had attended some training on antibiotic prescribing recently (2019–2020). 

There were almost an equal number of GPs that dispense (101–48.3%)) and those that do not dispense medicines (108–51.7%)) (Table 2). Physicians between the ages of 35–44 years of age (OR: 3.38; 95%CI: 1.15–9.88) and > 55 years (OR: 4.75; 95% CI 1.08–21), those prescribing more than 10 antibiotics per week (OR: 2.39; 95%CI: 1.15–4.95), and those in practice < 15 years (OR: 2.20; 95%CI: 1.08–4.51) were significantly more likely to prescribe antibiotics empirically. Male GPs (OR: 0.46; 95%CI: 0.23–0.90) and those in solo practices (OR: 0.29; 95%CI: 0.14–0.64) were significantly less likely to prescribe antibiotics empirically (Table 3). 

### 3.4. Environmental Factors Associated with Empiric Antibiotic Prescribing

Sixteen variables were tested (Table 4), with the full analysis in Table S1 in the Supplementary Material. Three factors, i.e., workload/time pressure, diagnostic uncertainty, and the use of a formulary, were significantly associated with empiric antibiotic prescribing. The lack of antibiotic prescribing guidelines was significantly less likely to influence empiric prescribing (Table 4 and Table S1). This is a concern as the current guidelines in South Africa do not encourage the empiric prescribing of antibiotics especially for viral infections including ARIs [95].

### 3.5. Physicians’ Knowledge and Empirical Antibiotic Usage

Physicians’ knowledge regarding their prescribing of antibiotics was assessed using ten questions (Table 5). The mean score of participating GPs was 7.58 (SD: 1.30) with a range between 3 and 10. The scores were centrally distributed with a median score of 8 (IQR: 7–9). 

Overall, 118 participating GPs (56.5%) had good knowledge regarding antibiotics, i.e., scored more than 8. Physicians with good self-reported knowledge were slightly less likely to prescribe antibiotics empirically but this was not significant (UOR: 0.96; 95% CI: 0.54–1.73). 

## 4. Discussion

We are aware of previous publications that have explored antibiotic prescribing rates for infections in ambulatory care across both sectors in South Africa. These include the management of ARIs, adherence rates to current antibiotic STGs and Essential Medicines Lists [14,37,41], as well as patient and physician’ attitudes towards the prescribing of antibiotics for ARIs [42,44]. However, we are currently unaware of any study that has been conducted in South Africa to assess key factors behind the high empiric prescribing of antibiotics for UTIs and ARIs in ambulatory care, which includes acute bronchitis, sinusitis and tonsillitis as well as COVID-19 infections. This is important given increasing concerns with AMR in South Africa [27].

Most doctors in the study were aware that AMR is a major public health threat. However, there was a high level of empiric prescribing for self-limiting conditions including ARIs and COVID-19 without evidence of bacterial co-infections, similar to studies in the public sector [14,44]. We found the strongest factor associated with empiric prescribing of antibiotics in the private sector was workload/time pressure. This was consistent with the findings from a number of other studies undertaken across countries and settings [96,97,98,99,100,101,102]. This could well be because under time pressures, physicians may find it more convenient to prescribe antibiotics rather than spending valuable time dissuading patients they do not need one. Such behaviour is exacerbated if patients expect an antibiotic for their ARI when visiting private GPs, enhanced by their perceived effectiveness for similar illnesses in the past [16,22,79,103]. An observational study in Norway also found that busier physicians prescribed antibiotics at a higher rate than their less busy colleagues [104]. However, others have failed to show a direct correlation between the duration of a consultation and the extent of prescribing of antibiotics for ARIs [105]. 

The empiric use of antibiotics was also more likely among GPs when there was diagnostic uncertainty, similar to the findings from recent studies from a number of other countries [106,107,108,109]. Prescribing antibiotics when there is uncertainty between possible viral or bacterial infections is exacerbated when laboratory tests are expensive, there is a lack of access to them as well as a lack of sensitive and cost-effective point-of-care testing equipment within LMIC settings [16,17,110,111]. GPs in South Africa and other LMICs also typically prescribe antibiotics to prevent complications and further infections. This is especially the case if there are extensive patient co-payments, high transport costs to healthcare centres and patients will lose income when taking time off work [16,42]. This needs to be taken into consideration when developing programmes to improve future antibiotic prescribing in ambulatory care across LMICs, which requires a systems approach to improve prescribing and patient safety [112].

GPs between the ages of 35–44 years, >55 years and in practice <15 years were also significantly more likely to prescribe antibiotics empirically in our study. The reason why GPs with less experience and younger GPs prescribe more antibiotics maybe because they find it more difficult to differentiate between viral or bacterial infections without pragmatic guidance, which could potentially include electronic decision support systems [52,108,113]. Alternatively, they are less confident dealing with demanding patients who expect an antibiotic. However, a study in Malta found older GPs exhibited habitual prescribing behaviour, relying on experience more than guidelines for their prescribing whereas younger GPs were more guideline-concordant [114]. In addition, the case mix may be different between younger and older GPs, with elderly patients more likely to visit more elderly GPs. We will be looking to explore this further.

Male GPs, those in solo practices, and where there was a lack of antibiotic prescribing guidelines in the practice, were associated with significantly less empiric prescribing of antibiotics. We have seen gender differences in other studies with female physicians more likely to prescribe antibiotics than their male counterparts [79,115,116]; this though is not always the case [117,118]. However, the findings that the availability of guidelines increased empiric prescribing of antibiotics is a concern. This is because we have seen that GPs in other studies who use guidelines are less likely to prescribe antibiotics empirically; however, guidelines must be easy to use, available and regularly referenced rather than sitting on shelves to achieve this [57,67,115,119,120,121]. These may be key factors here, and we will be exploring this further in future studies.

Overall, we postulate that the extensive empiric prescribing of antibiotics especially for viral infections, including acute tonsilitis, bronchitis, COVID-19 and coughs (Figure 2), could possibly be due to a number of factors. These include the potentially high number of patients seen by some GPs, time pressures, the lack of on-site testing, and a lack of regular follow-up of GP prescribing behaviour using agreed indicators (Table 1). In addition, in our study, less than 60% of GPs demonstrated good knowledge regarding antibiotics, which is similar to other studies in South Africa [38]. This is a concern as GPs with good knowledge were less likely to prescribe antibiotics empirically. This is similar to other studies where the higher the knowledge score, the less likely the physician is to prescribed antibiotics inappropriately [24,122]. In China as well, the higher the level of antibiotic knowledge, the greater the behavioural intention to prescribe less antibiotics, with less knowledge directly associated with the intention to prescribe antibiotics for patients with URTIs [123]. 

### Study Limitations

We are aware of a number of limitations with this study. Firstly, the questionnaire was purposely developed for this study without being validated. However, it was based on published studies combined with the considerable experience of the co-authors, similar to other approaches adopted by the co-authors [16,27,90,91,92,93,94], with a pilot study undertaken beforehand to enhance its robustness. The outcomes from the pilot study were subsequently incorporated into the principal study. Secondly, since GPs were invited online, this automatically excludes those GPs less familiar with the internet. Some GPs also did not fully complete the questionnaire for a variety of reasons including COVID-19 related reasons, which may have biased the findings. Alongside this, the actual sample size may have affected the findings of the regression analysis and the implications. 

Fourthly, we only targeted private GPs for this study for the reasons stated. However, we have made reference to a number of published studies discussing the prescribing habits of physicians working in the public ambulatory care system where appropriate. 

Furthermore, we were unable to ascertain whether the comments made were a true reflection of GPs’ opinions. However, this is a recognized drawback of all surveys of this nature. Finally, market research activities typically undertaken by GPs in a number of countries are usually remunerated as these are generally initiated by pharmaceutical companies. The lack of remuneration in this study may have resulted in doctors not truly focusing on the questions and giving the information without truly thinking of their replies. Despite these limitations, we feel the findings are robust given the number of GPs who participated providing future direction. 

## 5. Conclusions and Recommendations

A combination of environmental factors including poor access to affordable tests, workload/time pressure, prevention of serious complications and diagnostic uncertainty, GP age and in practice < 15 years, were associated with increased empiric prescribing of antibiotics in our study. Male GPs, those in solo practice, and a lack of prescribing guidelines also impacted on the empiric prescribing of antibiotic. These factors indicate that the social contexts are important underlying contributors to the empiric prescribing of antibiotics. 

There have been a number of activities across countries, including LMICs, to successfully improve the prescribing of antibiotics in ambulatory care. These typically centre on formative and educational initiatives, improving communication skills, as well as the availability and active dissemination of easy-to-use and up-to-date guidelines (Supplementary Material Table S2), with multifaceted interventions typically having a greater impact, similar to other disease areas [124,125,126]. We have seen high adherence rates to prescribing guidance in Stockholm, Sweden, and other countries where the guidance is trusted by GPs, readily available, and easy-to-use, with prescribing regularly monitored [127,128,129]. However, there must be consistency between the advice in national guidelines otherwise this causes uncertainty among prescribers [130]. The findings can be used by the private health insurers in South Africa to develop future educational needs specific to the South African context.

Alongside this, the insurance companies should develop indicators that can be agreed among all key stakeholders in South Africa to improve future antimicrobial prescribing in ambulatory care [131,132,133]. A number of indicators have been developed across countries to improve antibiotic prescribing in ambulatory care (Table 1), which provide a basis for the insurance groups in South Africa to take forward. In the future, including across Africa, these are increasingly likely to include agreed prescribing targets surrounding the Access’ and ‘Watch’ antibiotics including ‘Access’ to ‘Watch’ ratios in the AWaRe list, alongside adherence targets to AWaRe guidance, building on current activities (Table 1) [64,134,135,136]. These can subsequently be refined for the South African context. However, any indicator that is developed must be seen as acceptable to all key stakeholder groups, measurable, recorded in a reliable and consistent manner, robust, and sensitive to change (Table 1). In addition, cognizant of current patient record systems, e.g., whether just currently collecting data on medicines prescribed by individual GPs or able to add in diagnostic and co-morbidity data. Any subsequent prescribing indicators that are developed and agreed among key stakeholder groups can subsequently be implemented as part of ongoing antimicrobial stewardship programmes in ambulatory care in South Africa, mirroring developments in hospitals. This is in line with the South African National Action Plan for AMR [27,29]. The monitoring of adherence rates to prescribing guidance will be enhanced by the growing use of Apps across countries to monitor prescribing patterns including South Africa [60,137,138], linked where possible to a diagnosis. This is important as the impact of interventions can diminish if prescribing practices are not routinely monitored [69]. We will be following this up in future studies among ambulatory care physicians in South Africa.

Other potential approaches in the future include the introduction of ready access rapid diagnostic tests (point-of-care C-Reactive Protein Testing–POC-CRPT) in GP surgeries to help distinguish between bacterial and viral infections [110,139,140]. These tests can be undertaken in the doctors’ rooms while consulting, and we will be exploring this further.

## Figures and Tables

**Table 1:** Indicators used across countries to improve the prescribing of antibiotics in ambulatory care.

Guidelines/Contents	Content/Nature
Key attributes of quality indicators (adapted from [53,54,55,56])	**Clear**: clearly defined aspect of quality of care **Valid**: Measures what was intended **Acceptable**: to those being assessed and the assessors **Consequences**: desired outcomes set a priori **Unintended consequences**: minimised and implementation issues known **Attributable**: achievement of the aspect of care defined by an indicator should be 100% under the control of those being assessed **Evidence base**: underpinned by guidelines and peer-reviewed publications **Feasible**: valid and reliable consistent data are available and collectable **Reliable**: minimal measurement error, reproducible findings **Sensitive to change**: has the capacity to detect changes in the quality of care provided to discriminate between and within subjects **Predictive value**: has the capacity to predict quality of care outcomes **Relevance**: be in a priority area where there’s a recognized gap between actual and potential performance that needs addressing, e.g., improving antibiotic prescribing for ARIs and other self-limiting infections in ambulatory care
Potential indicators	Consumption of antibiotics based on agreed metrics, e.g., total number of Defined Daily Doses (DDDs), number of DDDs per patient encounters or per patient days, DDDs per 1000 inhabitants per day [57,58,59] Consumption of antibiotics based on agreed metrics, e.g., total number of Defined Daily Doses (DDDs), number of DDDs per patient encounters or per patient days, DDDs per 1000 inhabitants per day [57,58,59] Correct antibiotic, correct dose, correct duration of course prescribed and correct route of administration [46,57,60,61] The% of broad versus narrow antibiotics prescribed [59,62] The% of ‘Access’ versus ‘Watch’ or ‘Reserve’ antibiotics, with an initial target of 60% ‘AWaRe’ antibiotics [15,63,64] % prescribing targets for designated antibiotics including fluoroquinolones [16,59] Agreed ranges for prescribing antibiotics across indications in ambulatory care including ARIs [65,66] Agreed adherence rates to robust national guidelines including those for ARIs [46,57,61,67,68,69,70] Antibiotics should not be prescribed for viral infections as well as most self-limiting bacterial infections as they will not impact on symptom relief/outcomes [46,70,71,72] Antibiotics should not be prescribed for patients with ARIs within the first 3 days unless a good reason, which is documented in the patient’s notes, or withheld for 3 days [46,70,73]

NB: ARIs = Acute respiratory infections.

**Table 2:** Inclusion and exclusion criteria.

Variable	Criteria
Inclusion	GP practising exclusively in the private sector Willing to sign informed consent Practicing in the eThekwini District Belonging to one of the following medical associations/medical group: Medicross, Intercare, Medical Centre (Group practice), Durban IPA, Isiphingo IPA, Pinetown IPA and Durban South Doctors Guild Prescribed antibiotics in the past 6 months
Exclusion	GPs not practising exclusively in the public sector GPs not practising within the eThekwini district GPs not belonging to any of the following medical associations/medical group: Medicross, Intercare, Medical Centre (Group practice), Durban IPA, Isiphingo IPA, Pinetown IPA and Durban South Doctors Guild GPs not having prescribed an antibiotic within the last 6 months

**Table 3:** Demographic factors associated with empiric antibiotic prescribing.

Variable	Category	Empirical Antibiotic Prescribing (n;%)	Antibiotics Not Prescribed Empirically (n;%)	Total (%)	Unadjusted Odds Ratio (95%CI)	Adjusted Odds Ratio (95%CI)
Age	25–34	11 (5.3%)	13 (6.2%)	24 (11.5%)	Reference	
	35–44	56 (26.8%)	27 (12.9%)	83 (39.7%)	2.45 (0.97–6.18)	3.38 (1.15–9.88) *
	45–54	30 (14.4%)	33 (15.8%)	63 (30.1%)	1.07 (0.42–2.75)	2.78 (0.77–10.02)
	>55 years	19 (9.1%)	20 (9.6%)	39 (18.7%)	1.12 (0.41–3.11)	4.75 (1.08–21) *
Gender	Females	61 (29.2%)	26 (12.4%)	87 (41.6%)		
	Males	67 (32.1%)	55 (26.3%)	122 (58.4%)	0.35 (0.18–0.65) **	0.46 (0.23–0.90) *
Type of practice	Solo	64 (30.6%)	70 (33.5%)	134 (64.1%)	0.41 (0.22–0.77) **	0.29 (0.14–0.64) **
	Group	52 (24.9%)	23 (11.0%)	75 (35.9%)		
No. of consultations per day	<50	97 (46.4%)	75 (35.9%)	172 (82.3%)		
	>50	19 (9.1%)	18 (8.6%)	37 (17.7%)	0.83 (0.37–1.77)	1.04 (0.46–2.35)
Dispensing doctor	Yes	52 (24.9%)	49 (23.4%)	101 (48.3%)	0.73 (0.41–1.31)	1.17 (0.6–2.32)
	No	64 (30.6%)	44 (21.1%)	108 (51.7%)		
Estimated number of antibiotics prescribed in the last week	≤10	28 (13.4%)	30 (14.4%)	58 (27.8%)		
	>10	88 (42.1%)	63 (30.1%)	151 (72.3%)	1.50 (0.78–2.88)	2.39 (1.15–4.95) *
Years in private practice	≥15 years	35 (16.7%)	47 (22.5%)	82 (39.2%)	0.43 (0.23–0.78) *	0.44 (0.17–1.12)
	<15 years	81 (38.8%)	46 (22.0%)	127 (60.8%)		
Attended any training on antibiotic prescribing between 2019–2020	Yes	90 (43.1%)	65 (31.1%)	155 (74.2%)	1.49 (0.76–2.91)	1.29 (0.65–2.57)
	No	26 (12.4%)	28 (13.4%)	54 (25.8%)		

NB: %s are based on a total number of 209 participating GPs; * = *p* < 0.05; ** = *p* < 0.001.

**Table 4:** Designated Environmental Factors Associated with Empiric Antibiotic Prescribing and Adjusted Odds Ratios.

Variable	Adjusted Odds Ratio (95%CI)
To prevent serious complications	3.25 (1.20–8.81) *
Duration of symptoms	0.64 (0.28–1.45)
Patient clinical condition	0.42 (0.17–1.07)
Diagnostic uncertainty	3.15 (1.40–7.07) **
Type of disease	2.71 (0.65–11.41)
Presence of comorbidities	0.99 (0.46–2.11)
Patient expectation/request	0.66 (0.129–3.36)
Antimicrobial resistance concerns	0.74 (0.33–1.67)
Peers/colleague opinion	1.16 (0.50–2.70)
Workload/Time pressure	19.35 (2.73–137.19)
Medical aid formulary	2.39 (1.10–5.16) **
International Conferences	0.27 (0.11–0.65)
Lack of resources (access to microbiology laboratory)	3.11 (0.99–9.71)
Pharmaceutical representative	1.65 (0.66–3.98)
Lack of antibiotics prescribing guidelines	0.018 (0.001–0.18) **
Microbiologist advice	1.96 (0.89–4.3)

NB: * = *p* < 0.05; ** = *p* < 0.001; %s are based on a total number of 209 participating GPs.

**Table 5:** Knowledge assessment on antimicrobial usage.

Variable	Category	Empiric Antibiotics Use (n;%)	Antibiotics Not Prescribed Empirically (n;%)	Total (%)
Dosage reduction of antibiotics is necessary for patients with renal failure?	Yes (Correct)	108 (51.7%)	83 (39.7%)	191 (91.4%)
	No	8 (3.8%)	10 (4.8%)	18 (8.6%)
Should antibiotics be prescribed for non-febrile diarrhoea?	Yes (Incorrect)	26 (12.4%)	13 (6.2%)	39 (18.7%)
	No (Correct)	90 (43.1%)	80 (38.3%)	170 (81.4%)
Antibiotics can be used for bacteria pneumonia (including one of the following symptoms: chest in-drawing or stridor, fast breathing).	YES (Correct)	106 (50.8%)	84 (40.2%)	190 (90.9%)
	NO (Incorrect)	10 (4.8%)	9 (4.3%)	19 (9.1%)
Do antibiotics reduce the duration and the occurrence of complications of upper respiratory tract infections?	YES (Incorrect)	47 (22.5%)	49 (23.4%)	96 (45.9%)
	NO (Correct)	69 (33.0%)	44 (21.1%)	113 (54.1%)
Is amoxicillin safe to use in pregnancy?	YES (Correct)	102 (48.8%)	83 (39.7%)	185 (88.5%)
	NO (Incorrect)	14 (6.7%)	10 (4.8%)	24 (11.5%)
Methicillin resistant staphylococcus aureus is resistant to beta-lactam antibiotics?	YES (Correct)	81 (38.8%)	72 (34.4%)	153 (73.2%)
	NO (Incorrect)	35 (16.7%)	21 (10.0%)	56 (26.8%)
Which of the following antibiotics crosses the blood–brain barrier?	Clindamycin (Incorrect)	60 (28.7%)	33 (15.8%)	93 (44.5%)
	Vancomycin (Incorrect)			
	Ceftriaxone (Correct)	44 (18.2%)	72 (34.4%)	116 (55.5%)
Methicillin resistant staphylococcus aureus is susceptible to which antibiotics?	Amoxicillin-clavulanic acid (Incorrect)	57 (27.3%)	35 (16.7%)	67 (32.1%)
	Ceftriaxone (Incorrect)			11 (5.3%)
	Cefotaxime (Incorrect)			14 (6.7%)
	None of these antibiotics (Correct)	59 (28.2%)	58 (27.8%)	117 (56.0%)
Use of broad spectrum antibiotics when a narrow spectrum antibiotics (equally effective) are available can increase antibiotics resistance	YES (Correct)	110 (52.6%)	6 (2.9%)	116 (55.5%)
	NO (Incorrect)	89 (42.6%)	4 (1.9%)	93 (44.55%)
Patients’ non-compliance to antibiotics drives antibiotics resistance	YES (Correct)	110 (52.6%)	6 (2.9%)	116 (55.5%)
	NO (Incorrect)	81 (38.8%)	12 (5.7%)	93 (44.5%)

NB: %s are based on a total number of 209 participating GPs.

## Data Availability

Additional data is available on reasonable request from the corresponding author.

## Supplementary Materials

The following supporting information can be downloaded at: https://www.mdpi.com/article/10.3390/antibiotics11101423/s1, Table S1: Designated Environmental Factors Associated with Empiric Antibiotic Prescribing. Table S2: Activities introduced across countries, especially LMICs, to improve antimicrobial prescribing and their impact. References [141,142,143,144,145,146,147,148,149,150,151,152,153,154,155] in Supplementary Tables.
