# Supplementary material for: Determinants of the Empiric Use of Antibiotics by General Practitioners in South Africa: Observational, Analytic, Cross-Sectional Study
Source: Antibiotics (Basel). 2022 Oct 17;11(10):1423. doi: 10.3390/antibiotics11101423 (PMC9598257; doi:10.3390/antibiotics11101423)
Supplement: Supplementary file 1 [file antibiotics-11-01423-s001.zip › antibiotics-1926633-supplementary.pdf]

Supplementary Tables

Table S1: Designated Environmental Factors Associated with Empiric Antibiotic Prescribing.

Variable	Category	Antibiotics prescribed empirically (n; %)	Antibiotics not prescribed empirically (n; %)	Total (%)	Unadjusted odds ratio (95% CI)	Adjusted Odds Ratio (95% CI)
To prevent serious complications	Very often/often	102 (48.8%)	14 (6.7%)	116 (55.5%)	4.01 (1.89-8.73) **	3.25 (1.20-8.81) *
	About half the time/ Rarely/ Almost never	60 (28.7%)	33 (15.8%)	93 (44.5%)		
Duration of symptoms	Very often/often	78 (37.3%)	38 (18.2%)	116 (55.5%)	1.92 (1.06-3.51) *	0.64 (0.28-1.45)
	About half the time/ Rarely/ Almost never	48 (23.0%)	45 (21.5%)	93 (44.5%)		
Patient clinical condition	Very often/often	91 (43.5%)	25 (12.0%)	116 (55.5%)	0.77 (0.351-1.60)	0.42 (0.17-1.07)
	About half the time/ Rarely/ Almost never	77 (36.8%)	16 (7.7%)	93 (44.5%)		
Diagnostic uncertainty	Very often/often	65 (31.1%)	51 (24.4%)	116 (55.5%)	4.65 (2.42-9.10) **	3.15 (1.40-7.07)**
	About half the time/ Rarely/ Almost never	20 (9.6%)	73 (34.9%)	93 (44.5%)		
Type of disease	Very often/often	111 (53.1%)	5 (2.4%)	116 (55.5%)	4.27 (1.29-15.54)*	2.71 (0.65-11.41_
	About half the time/ Rarely/ Almost never	78 (37.3%)	15 (7.2%)	93 (44.5%)		
Presence of comorbidities	Very often/often	65 (31.7%)	51 (24.4%)	116 (55.5%)	1.05 (0.58-1.89)	0.99 (0.46-2.11)
	About half the time/ Rarely/ Almost never	51 (24.4%)	42 (20.1%)	93 (44.5%)		
Patient expectation/request	Very often/often	17 (8.1%)	99 (47.3%)	116 (55.5%)	1.82 (0.70-5.13)	0.66 (0.129-3.36)
	About half the time/ Rarely/ Almost never	8 (3.8%)	85 (40.7%)	93 (44.5%)		

Antimicrobial resistance concerns	Very often/often	87 (41.6%)	29 (13.9%)	116 (55.5%)	1.65 (0.87-3.14)	0.74 (0.33-1.67)
	About half the time/ Rarely/ Almost never	60 (28.7%)	33 (15.8%)	93 (44.5%)		
Peers/colleague opinion	Very often/often	50 (23.9%)	66 (31.6%)	116 (55.5%)	1.52 (0.87-2.78)	1.16 (0.50-2.70)
	About half the time/ Rarely/ Almost never	31 (14.8%)	62 (29.7%)	93 (44.5%)		
Workload/Time pressure	Very often/often	22 (10.5%)	94 (45.0%)	116 (55.5%)	5.21 (1.67-21.46)**	19.35 (2.73-137.19)
	About half the time/ Rarely/ Almost never	4 (1.9%)	89 (42.6%)	93 (44.5%)		
Medical aid formulary	Very often/often	88 (42.1%)	39 (18.7%)	127 (60.8%)	4.35 (2.31-8.23)**	2.39 (1.10-5.16)**
	About half the time/ Rarely/ Almost never	28 (13.4%)	54 (25.8%)	82 (39.2%)		
International Conferences	Very often/often	22 (10.5%)	94 (45.0%)	116 (55.5%)	0.43 (0.94-1.04)	0.27 (0.11-0.65)
	About half the time/ Rarely/ Almost never	33 (15.8%)	60 (28.7%)	93 (44.5%)		
Lack of resources (access to microbiology laboratory)	Very often/often	27 (12.9%)	89 (42.6%)	116 (55.5%)	2.26 (1.00-3.37)	3.11 (0.99-9.71)
	About half the time/ Rarely/ Almost never	11 (5.3%)	82 (39.2%)	93 (44.5%)		
Pharmaceutical representative	Very often/often	53 (25.3%)	63 (30.1%)	116 (55.5%)	3.77 (1.91-7.61)**	1.65 (0.66-3.98)
	About half the time/ Rarely/ Almost never	17 (8.1%)	76 (36.4%)	93 (44.5%)		

Lack of antibiotics prescribing guidelines	Very often/often	3 (1.4%)	113 (54.1%)	116 (55.5%)	0.25 (0.04-1.04)	0.018 (0.001-0.18)**
	About half the time/ Rarely/ Almost never	9 (4.3%)	84 (40.2%)	93 (44.5%)		
Microbiologist advice	Very often/often	80 (38.4%)	36 (17.2%)	116 (55.5%)	2.00 (1.09-3.66)*	1.96 (0.89-.43)
	About half the time/ Rarely/ Almost never	49 (23.4%)	44 (21.1%)	93 (44.5%)		

NB: * = p<0.05; ** = p<0.001; %s are based on a total number of 209 participating GPs

Table S2 – Activities introduced across countries, especially LMICs, to improve antimicrobial prescribing and their impact

Author, Country, Year	Intervention and impact	Impact
**High-income countries**		
6 European countries (2013) [141]	Principally education –<br>Allocation to usual care for patients with ARIs, training of GPs in the use of a C-reactive protein (CRP) testing at point of care and in enhanced communication skills (via the internet), or both interventions combined	• CRP training – antibiotic prescribing rates were lower than controls (33% vs 48%)<br>• Enhanced-communication training - antibiotic prescribing rates were lower than controls (36% vs 45%)<br>• The combined intervention led to the greatest reduction in prescribing rates (combined 0·38, 0·25-0·55, p<0·0001).
Norway (2013) [142]	Principally education:<br>• Intervention groups - two visits by peer academic detailers, the first presenting national clinical guidelines for antibiotic use in ARIs, the second discussing each GP's current antibiotic prescribing profile in addition to one day seminars<br>• The control arm received targeted prescribing practice for older patients (continual medical education)	Among the intervention group:<br>• a reduction (odds ratio 0.72) in the prescribing of antibiotics for ARIs compared with the controls<br>• a reduction in the odds (0.64, 0.49 to 0.82) for the prescribing a non-penicillin V antibiotic when an antibiotic was issued
UK (2014) [143]	Principally education - Electronically delivered decision support tools were evaluated to reduce antibiotic prescribing for patients with ARIs in primary care (remotely installed and accessible during the consultations) versus a control group (no support tools)	The decision support tool was used less than anticipated, varying though among the included practices.<br><br>In the intervention group:<br>• There was a 1.85% reduction in the proportion of consultations with antibiotics prescribed<br>• These was a 9.69% reduction in the rate of antibiotic prescribing for ARIs with fewer prescriptions per 1,000 patient-years<br>• There were no adverse events
USA (2013) [72]	Principally education as part of a three-arm, cluster-randomized trial:<br>• The printed intervention group received decision support (printed material) for acute cough illness<br>• The computerized intervention group received decision support through an electronic medical record-based strategy<br>• Control arm<br>Both intervention groups also received provider education and feedback as well as patient education brochures	• In the printed material group, antibiotic prescribing decreased from 80.0% to 68.3%<br>• In the computerized intervention sites a decrease in antibiotic prescribing from 74.0% to 60.7%<br>• A slight increase in the control sites from 72.5% to 74.3%<br>• Both interventions significantly different from the control group but not from each other

USA (2016) [144]	Principally education combined with monitoring in a controlled study involving 3 interventions alone or in combination to reduce inappropriate antibiotic prescribing for ARIs in primary care<br>The three interventions included:<br>• suggested alternatives – typically suggesting nonantibiotic treatments electronically<br>• accountable justification - prompting clinicians to enter free-text justifications for their prescribing into patients' electronic health records<br>• peer comparisons – allowing GPs compared their prescribing rates with the top performers	• Control group - mean antibiotic prescribing decreased from 24.1% of patients at the start to 13.1% 18 months later<br>• Suggested alternative group - mean antibiotic prescribing decreased from 22.1% to 6.1% of patients after 18 months<br>• Accountable justification group – mean antibiotic prescribing decreased from 23.2% to 5.2% of patients after 18 months<br>• Peer comparison group - mean antibiotic prescribing decreased from 19.9% to 3.7% of patients after 18 months<br>• All inreventions significantly reduced antibiotic prescribing versus the control arm<br>• There were no statistically significant differences between the interventions, or when these were combined
**LMICs**		
Chowdhury et al, Bangladesh, 2007 [145]	3-arm study principally educational study involving 3 groups:<br>• Group I – education surrounding STGs and subsequent auditing of prescribing<br>• Group-II just education surrounding STGs<br>• Group-III - control (no intervention)	• The prescribing of unnecessary antibiotics to treat ARI was significantly reduced ($p<0.01$) compared to the pre-intervention period in Group I, with the average reduction in antibiotic use (patient encounters) of 23.7% in Group I and 15.2% in Group II<br>• When factoring in the Control group (Group III) – there was a 15.2% reduction in antibiotic use in Group I and 6.9% in Group II
Yip et al, China, 2014 [146]	• Principally Economics<br>• In Ningxia Province, a randomized study was undertaken to evaluate the impact of capitation with the introduction of pay-for-performance measures on subsequent antibiotic prescribing, health spending, outpatient visit volumes, and patient satisfaction	• Approximately 15% reduction in antibiotic prescriptions following the implementation of pay-for-performance measures<br>• A small reduction in total spending per visit
Wei et al, China, 2019 [18]	• Principally education involving multiple interventions<br>• Interventions comprised of: clinical guidelines, monthly prescribing review meetings, training in doctor–patient communication skills, and the provision of education materials for caregivers	• A 49% reduction in the prescribing of antibiotics for children with URTIs after 6 months in the intervention arm - having adjusted for patient and prescribing doctor covariates<br>• The reductions persisted after 18 months but at a lower rate (-36%)<br>• Key factors for sustaining the reduction in antibiotic prescribing included physicians' improved knowledge and communication skills combined with prescription review meetings

India (2018) [147]	• Principally education to reduce the rate of inappropriate prescribing of antibiotics for URTIs<br>• Education consisted of a repeated process of audit and feedback combined with interactive training sessions, one-to-one case-based discussion, antibiotic guideline development and coding updates	• Antibiotic prescribing reduced from 62.6% of patients prescribed antibiotics to 7.2%<br>• This was combined with an increase in the documentation of examination findings from 52.7% to 95.6% of patients justifying the treatment approach
Korom et al, Kenya, 2017 [148]	• Multiple interventions principally surrounding education.<br>• These included:<br>o The formal introduction of a clinical practice guideline<br>o Introduction of peer-to-peer chart reviews<br>o Peer-reviewed publication describing local antimicrobial resistance patterns<br>• The interventions were undertaken by trained clinical officers	• Adherence to guideline-recommended antibiotics improved significantly - from 19% at baseline to 68% following all interventions (X2 = 150.7, $p < 0.001$)<br>• An outcome of composite quality scores improved significantly from an average of 2.16 to 3.00 on a five-point scale ($t = 6.58$, $p < 0.001$)<br>• The interventions had different effects at different clinical sites - possibly reflecting differences in clinical officers and their activities<br>• Provider age was not a significant factor in subsequent changes in prescribing habits
Teng et al, Malaysia, 2006 [149]	Education – Including academic detailing from the resident family medicine specialist combined with an information leaflet	• A reduction in antibiotic prescribing rates from to 11.0% of patients post-intervention from 14.3% pre-intervention (RR 0.77, 95% CI 0.72 to 0.83)<br>• Reduction in URTI-specific antibiotic prescribing rates to 16.6% post-intervention from 27.7% of patients pre-intervention (RR 0.60, 95% CI 0.54 to 0.66)
Tay et al, Malaysia, 2019 [150]	Principally Education involving educational toolkits<br>These included:<br>• A training module for HCPs on ARIs and acute diarrhoea involving one-hour educational sessions covering diagnostic criteria and treatment decision pathways<br>• Educational posters in Malay and English in the waiting area and consultation rooms<br>• Multimedia educational videos in the waiting areas<br>• Physician reminders	• Appreciable reduction in antibiotic prescribing:<br>o ARIs down from 29.1% to 13.7%<br>o Acute diarrhea down from 11.2% to 6.7%
Shrestha et al, Nepal, 2006 [151]	• Principally Education<br>This included 5 days of training on the adapted Practical Approach to Lung Health (PAL) guidelines and their use	• Appropriate prescribing of antibiotics and adherence to guidelines improved<br>• However, this was not statistically significant versus other areas such as polypharmacy and generic prescribing rates
Kafle et al, Nepal, 2009 [152]	• Principally Education<br>• This included:	• In children under five, there was a significant improvement in use of antimicrobials in diarrhea

	o Supervision/ monitoring involving periodic visits by district supervisors to 41 primary health care (PHC) facilities<br>o Small-group training among prescribers<br>o Peer-group discussions<br>o Self-assessment of the data presented	• There was also a significant improvement in the prescribing of antibiotics for URTIs without pneumonia
Hamilton et al, Sierra Leone, 2018 [69]	• Principally education<br>• Provision of an empirical antimicrobial guideline introduced via a number of different methods<br>• These included:<br>o One-to-one feedback meetings<br>o Announcements of the guidelines in general meetings<br>Printed copies of the guideline in each outpatient room	• After the first cycle, the choice of appropriate antimicrobial improved to 85% and the correct antibiotic, dose and course-length to 53%<br>• Unfortunately, 2 months later the rates reduced to 65% and 43%, respectively following lack of follow-up<br><br>Implementing a guideline can be effective at improving appropriate antibiotic prescription; however, repeated measures are needed for sustainable change
Awad et al, Sudan, 2006 [153]	• Principally education<br>• Twenty health centers in Khartoum State were randomly assigned to receive either:<br>o No intervention<br>o Audit and feedback<br>o Audit and feedback plus a seminar on antibiotic prescribing<br>Audit and feedback combined with academic detailing	• Significant reduction in the mean number of physician encounters with an antibiotic prescribed by 6.3 and 7.7 ($p<0.001$) at 1 and 3 months post-intervention, respectively<br>• Significant reduction in the mean number of encounters where antibiotics were inappropriately prescribed ($p<0.001$)<br>• However, reducing the number of activities/ interventions reduced their overall impact
Boonyasiri et al, Thailand, 2014 [154]	• Principally Education including:<br>o Training HCPs on the rational use of antibiotics<br>o Introduction of clinical practice guidelines<br>o Potential for throat swabs (stool cultures for acute diarrhea)<br>Printed brochures for patients/ relatives in waiting rooms containing likely aetiology as well necessity and harm of antibiotics for ARIs and acute diarrhea	• The multifaceted programme resulted in:<br>o Limited prescribing of antibiotics for ARIs (13.0%) and for acute diarrhea (19.1%)<br>• Clinical responses on day 3 after receiving care showed that more than 97% of the patients who received antibiotics/ those who did not receive antibiotics were cured or improved
Hoa et al Vietnam 2017 [155]	Multi-faceted educational intervention targeting health-care-providers' knowledge, practical competences and prescribing of antibiotics for ARI.	• Knowledge improved in the intervention group for patients with ARIs by 28%, antibiotic use for mild ARIs by 15% and severe ARIs by 14%<br>• Practical competence for patients with mild ARIs cimproved in the intervention and control groups by 20% and 11% respectively

		• The practice regarding the prescribing of antibiotics for patienst with mild ARIs improved by 28% in the intervention group
